# Supplementary material for: The Impact of Long-Term Macrolide Exposure on the Gut Microbiome and Its Implications for Metabolic Control
Source: Microbiol Spectr. 2023 Jun 22;11(4):e00831-23. doi: 10.1128/spectrum.00831-23 (PMC10433835; doi:10.1128/spectrum.00831-23)
Supplement: Supplemental file 1 — Supplemental material. Download spectrum.00831-23-s0001.pdf, PDF file, 1.7 MB [file spectrum.00831-23-s0001.pdf]

SUPPLEMENTARY MATERIAL

Long-term macrolide exposure influences metabolic control through alteration of the gut microbiome

Jocelyn M Choo[1,2], Alyce Martin[3], Steven L Taylor[1,2], Emily Sun[3], Fredrick M Mobegi[1,2], Tokuwa Kanno[4], Alyson Richard[1,2], Lucy D Burr[5,6], Stevie Lingman[5], Megan Martin[5], Damien J Keating[3,7], A James Mason[4], Geraint B Rogers[1,2]*

Human cohort

Healthy adults were included to the study if they had not taken any antibiotics in the preceding three months, or macrolide antibiotics in the preceding 12 months, had not experienced respiratory illness in the preceding month and a non-smoker. Subjects were excluded from the study if they had illicit drug use, current use of medications including medications prolonging QT interval, immunomodulators and corticosteroids, had a chronic lung disease (including asthma), have a current or previous medical history including cardiac disease, active arrhythmia, hepatic disease, active malignancy, immunosuppression and allergy to macrolide antibiotics, women who are pregnant or have been pregnant in the preceding six months, breast-feeding individuals (pregnancy category C) and if they have significant hospital contact or are based within a healthcare setting. Safety assessments, including screening based on medical history and an electrocardiogram (ECG) for long QTc, were performed on subjects immediately prior to the commencement of antibiotics (baseline), week one and week four of macrolide treatment.

A twice-daily regimen was chosen for both macrolide groups to maximise adherence. Azithromycin and erythromycin capsules (Alphapharm Research Ltd, Queensland, Australia)

were prepared by Mater Health Pharmacy. Subjects were allocated (1:1) to azithromycin or erythromycin using concealed random allocation from a computer-generated random numbers table with permuted blocks of 2 or 4. All study personnel and investigators involved in sample processing and data entry were blinded to the treatment assignment, however the patients were not. This was felt acceptable as all outcomes were microbiological, not patient-orientated, and therefore not subject to bias. Primary outcomes and sample size calculation are as previously detailed (Burr et al., 2022).

To further assess whether the sample sizes was sufficient for gut microbiome analysis, power calculation was also performed based on previous human studies that assessed macrolide-associated effects on the gut microbiome, which includes a short-term (3-day) course of 10mg/kg azithromycin in children(Wei et al., 2018), and a single course of 20mg/kg azithromycin treatment in children aged up to 60-months with gut microbiome analysis performed five days after the antibiotic treatment (Doan et al., 2017). The effect size calculated for changes in gut microbial diversity were 0.64 and 0.73, respectively. Therefore, a sample size of at least 19 is required to achieve a type 1 error rate of 5% and power of 0.9.

Mice studies

Female C57BL/6 mice were bred at the SAHMRI Bioresources animal facility, while female germ-free C57BL/6 mice were obtained from the Translational Research Institute (Queensland, Australia). All mice were acclimatized for a period of two weeks prior to study commencement and maintained at 22°C ± 2°C under a 12-hr light-dark cycle (lights on between 0700 and 1900) throughout the study period. Faecal pellet collection prior to antibiotic treatment (20mg/kg erythromycin ethylsuccinate, supplemented in drinking water) and at day 90 was performed by placing mice into individual clean cages. Faecal pellets were collected into a 1.5mL Eppendorf

tube using sterile toothpicks and stored at -80°C until use. At the end of each study, mice were humanely killed by carbon dioxide inhalation.

The dosage of erythromycin used in mice studies were determined based on a correction factor (K_m) to convert between dosages in animal studies and the human equivalent dose (HED) (Nair et al., 2016), which take into account differences in body size and drug clearance rates. The correction factor (K_m) was determined based on the ratio of the calculated average body weight (kg) to body surface area (m^2) between species. Using this calculation, the resulting correction factor (Km) value for human to mice dose conversion is reported to be 12.3, which dosage is divided by 10 as a safety measure.(Nair et al., 2016) To determine the corresponding dose for low-dose erythromycin studies in mice, we performed calculations based on an average human BMI of 60kg, 800mg/day of erythromycin ethylsuccinate in humans, a correction factor of 12.3 and a safety dose of 0.1. In accordance with the calculation (800mg/60kg) * 12.3 * 0.1, the equivalent dosage for low-dose erythromycin in mice was estimated to be 16.4mg/kg. Therefore, a dosage at 20mg/kg erythromycin ethylsuccinate (20% surplus) was administered to account for potential variation in drinking water intake.

Bioinformatics and statistical analysis

Using shotgun metagenomics sequencing on human faecal samples, high quality reads were obtained at an average ± standard deviation of 42.2 ± 8.9 million reads per sample and a base call Q30 of 88.6 ± 1.0%. Quality trimming and adapter removal were performed on paired-end reads using Trimmomatic v0.39 (Truong et al., 2015), based on the parameters TruSeq3-PE.fa:3:30:10 MINLEN:36 HEADCROP:10, followed by quality checking of the sequence reads using FASTQC (v0.11.9). Contaminating human sequence reads were removed based on the human reference genome (GRCh38) using Bowtie2 (v2.3.5.1) with default parameters.

Downstream analysis of samples was performed according to previous parameters (Mobegi et al., 2020), resulting in 22.3 ± 2.9 million quality-filtered reads of at least 138 nucleotides and a Q30 score of ≥20.

To calculate between-sample dissimilarity for microbiota composition analysis, Bray Curtis distances were computed on square root-transformed species relative abundances for shotgun metagenomics data, and weighted Unifrac distances were computed on genus-level relative abundances using QIIME2 for 16S rRNA amplicon sequencing data.

Measurements of host physiology

Faecal pH was measured using a FE20 FiveEasyTM pH meter (Mettler-Toledo AG, Schwerzenbach, Switzerland). Human faecal samples were diluted in 2x volume deionized water (Choo et al., 2021a), and mouse faecal pellets were diluted in 9x deionized water, prior to determination of pH (Choo et al., 2021b). Gastrointestinal transit time in mice was assessed using carmine red dye (3% w/v solution in 0.5% methylcellulose) (Sigma-Aldrich, St Louis, USA). Mice received an oral gavage of 150 µL of carmine red dye solution and faecal output was monitored every 30 min or upon spontaneous passing of faecal pellets (Dey et al., 2015). Time from gavage to appearance of bright red dye in faecal pellets was recorded as gut transit time. Mouse caecum weight was recorded at the end of the study.

ELISA analysis

Serum cytokine and hormone levels were quantitated using a combination of commercially available multiplex immunoassay panels and enzyme-linked immunosorbent assays (ELISA). Specifically, GM-CSF, IFNγ, IL-1β, IL-4, IL-5, IL-6, IL-7, IL-8, IL-10, IL-12(p70), IL-13, IL-17A and TNFα were assayed using the Millipore human high sensitivity T cell panel

(MPHSTCMAG28SK13); IL-15, MCP-1 and IL-9 were assayed using the Millipore Human Cytokine/Chemokine Panel I (MPHCYTOMAG60K03); HGF, FGF2 and leptin were assayed using the Millipore Human Circulating Cancer Panel 1 (MPHCCBP1MAG58K03); adiponectin was assayed using the Millipore Human Adipokine Panel 1 (MPHADK1MAG61K01); C-peptide, active ghrelin, total GIP, total GLP-1, glucagon, insulin and total PYY were assayed using the Millipore Human Metabolic Hormone Panel (MPHMHEMAG34K07). Serum C-reactive protein (CRP), serotonin (5-HT) and Fibroblast Growth Factor 19 (FGF19) were quantitated using commercially available ELISA kits (MPCYT298, Merck Millipore; BA E-5900, LDN; and EHFGF19, Thermofisher Scientific, respectively). Serum glucose level was assayed using a commercially available hexokinase kit (GAHK20, Sigma Aldrich) and lipopolysaccharide (LPS) levels were quantitated using a Pierce™ LAL Chromogenic Endotoxin Quantitation Kit (Thermofisher Scientific). Serum glucose level was assayed using a commercially available hexokinase kit (GAHK20, Sigma Aldrich). For the Human Metabolic Hormone Panel, serum sample volume used for each well was doubled to 50 μL to enable better detection of the analytes. For the serotonin assay, serum was diluted (1:1000) before they were added to the sample wells. All sample were run in duplicates and all quality controls fell within the expected ranges.

SUPPLEMENTARY INFORMATION

Table S1. Total faecal bacterial load in healthy adults following exposure to macrolides for 4 weeks

	Antibiotic	Baseline	ABX	p
**Total bacterial load**	Erythromycin	8.9×10^6 (6.8×10^6 – 1.4×10^7)	9.6×10^6 (7.5×10^6 - 1.2×10^7)	0.625
	Azithromycin	1.2×10^7 (5.8×10^6 – 1.7×10^7)	9.4×10^6 (6.0×10^6 - 1.2×10^7)	0.375
**Faecal pH**	Erythromycin	6.7 (± 0.64)	7.1 (± 0.48)	0.131
	Azithromycin	6.8 (± 0.27)	7.0 (± 0.46)	0.193

Total bacterial load of faecal samples (based on copies of 16S rRNA gene/μL) is represented as the median and interquartile ranges. Faecal pH is represented as mean and standard deviation. ABX refers to the 4-week duration of macrolide treatment. The p-values were determined based on the Wilcoxon test.

Table S2. Microbial species that were significantly altered in healthy adults following exposure to erythromycin and/or azithromycin for 4 weeks.

Microbial species	Erythromycin (ERY)		Azithromycin (AZM)		Log2 fold change	p	FDR
	Baseline	ABX	Baseline	ABX			
Actinomyces johnsonii	0 (0 - 0.001)	0.0094 (0 - 0.0203)	0 (0 - 0)	0.0363 (0.0091 - 0.0808)	4.00	<0.001	0.031
Actinomyces sp. ICM47	0.0136 (0.0002 - 0.0329)	0 (0 - 0)	0.0008 (0 - 0.0023)	0 (0 - 0)	-2.41	0.01	0.151
Actinomyces sp. oral taxon 170	0 (0 - 0)	0 (0 - 0.0018)	0 (0 - 0)	0.0097 (0.0057 - 0.0172)	3.80	0.003	0.06
Actinomyces sp. oral taxon 448	0 (0 - 0.0041)	0 (0 - 0.0018)	0 (0 - 0)	0 (0 - 0)	0.00	0.035	0.231
Bifidobacterium adolescentis	0.4822 (0 - 5.8421)	0 (0 - 0)	1.7836 (0.068 - 8.0509)	0 (0 - 0)	-12.30	0.002	0.05
Bifidobacterium bifidum	0.8392 (0 - 3.7463)	0 (0 - 0)	0 (0 - 0)	0 (0 - 0)	0.00	0.022	0.207
Bifidobacterium longum	2.3024 (0.4312 - 5.5853)	0 (0 - 0)	1.8175 (0.3471 - 2.5887)	0 (0 - 0)	-13.69	<0.001	0.025
Colinsella stercoris	0.0043 (0.0029 - 0.0161)	0 (0 - 0)	0.0065 (0.0013 - 0.0157)	0.0005 (0 - 0.0036)	-3.29	0.012	0.151
Eggerthella lenta	0.1342 (0.03 - 0.3012)	0.6233 (0.1824 - 1.013)	0.0049 (0 - 0.0804)	0.1496 (0.0541 - 0.3808)	2.08	<0.001	0.025
Barnesiella intestinihominis	0.3443 (0.0414 - 0.7214)	0 (0 - 0)	0.1816 (0.0626 - 0.334)	0 (0 - 0.1179)	-8.32	0.042	0.242
Odoribacter splanchnicus	0.0838 (0.0097 - 0.0943)	0.0138 (0 - 0.1149)	0.058 (0.0345 - 0.2636)	0 (0 - 0.0213)	-1.08	0.032	0.231
Gemella sanguinis	0 (0 - 0.0033)	0 (0 - 0.0178)	0 (0 - 0)	0.0045 (0 - 0.0142)	0.00	0.011	0.151
Streptococcus australis	0 (0 - 0.0037)	0 (0 - 0.0134)	0 (0 - 0)	0 (0 - 0.0064)	0.00	0.018	0.199
Streptococcus mitis	0 (0 - 0)	0 (0 - 0.0048)	0 (0 - 0)	0.001 (0 - 0.004)	0.00	0.021	0.201
Streptococcus salivarius	0.2727 (0.2052 - 0.4549)	0.127 (0.0656 - 0.1737)	0.1688 (0.0474 - 0.6003)	0.1002 (0.0472 - 0.1905)	-0.97	0.04	0.242
Clostridium sp. CAG:242	0 (0 - 0.0021)	0 (0 - 0)	0 (0 - 0.0642)	0 (0 - 0)	0.00	0.036	0.231
Eubacterium ramulus	0.1808 (0.132 - 0.5208)	0.7781 (0.1109 - 1.0168)	0.3359 (0.1856 - 0.3818)	0.5675 (0.3952 - 1.0357)	0.77	0.005	0.099
Blautia hydrogenotrophica	0.0005 (0 - 0.0032)	0 (0 - 0.0078)	0.0118 (0 - 0.0755)	0.0615 (0 - 0.229)	0.00	0.038	0.235

Blautia obeum	1.4519 (1.0487 - 2.2521)	2.7135 (1.5752 - 5.1705)	2.2345 (1.8939 - 3.1247)	2.1599 (1.6468 - 3.3657)	0.57	0.048	0.267
Blautia sp. CAG:257	0 (0 - 0.0002)	0 (0 - 0.0224)	0 (0 - 0.0007)	0 (0 - 0.0132)	0.00	0.021	0.201
Ruminococcus gnavus	0.0062 (0 - 0.0617)	0.1439 (0.0055 - 0.9014)	0.0131 (0.002 - 0.0402)	0.1073 (0.0071 - 0.2309)	1.94	<0.001	0.031
Coprococcus catus	0.321 (0.1635 - 0.6035)	0.5981 (0.2763 - 0.7819)	0.391 (0.1973 - 0.6406)	0.267 (0.1578 - 0.6542)	0.49	0.05	0.267
Coprococcus comes	0.9453 (0.5085 - 2.0452)	2.6933 (0.8533 - 4.8249)	1.5561 (1.3032 - 2.781)	2.9527 (1.7707 - 3.7385)	0.85	<0.001	0.03
Dorea longicatena	2.4373 (1.9778 - 3.0904)	3.2279 (1.9409 - 7.1334)	3.2959 (2.4782 - 4.9055)	3.9336 (2.6701 - 4.586)	0.36	0.029	0.231
Eisenbergiella tayi	0 (0 - 0.0009)	0.0006 (0 - 0.0047)	0 (0 - 0.0004)	0 (0 - 0.0101)	0.00	0.029	0.231
Fusicatenibacter saccharivorans	3.4095 (1.6856 - 6.1229)	5.5685 (2.2709 - 8.9846)	3.3815 (2.1581 - 6.3964)	5.9545 (3.2068 - 7.4802)	0.39	0.012	0.151
Clostridium bolteae	0 (0 - 0.0008)	0 (0 - 0.004)	0 (0 - 0.0011)	0 (0 - 0)	0.00	0.042	0.242
Roseburia inulinivorans	0.4608 (0.1751 - 0.7264)	0.038 (0.0064 - 0.1433)	0.1493 (0.0337 - 0.27)	0.2234 (0.0446 - 0.5062)	-0.10	0.457	0.733
Anaeromassilibacillus sp. An250	0.008 (0.0017 - 0.0267)	0.0271 (0.0081 - 0.0971)	0.007 (0.0005 - 0.0164)	0.0044 (0.0008 - 0.0122)	0.77	0.088	0.304
Flavonifractor plautii	0.04 (0.0128 - 0.095)	0.052 (0.0348 - 0.5552)	0.0583 (0.0107 - 0.0965)	0.0803 (0.0407 - 0.1377)	0.82	0.011	0.151
Ruminococcus bicirculans	0.6329 (0.2281 - 1.1142)	0 (0 - 0.2344)	0.3808 (0.1485 - 2.039)	0 (0 - 0.8848)	-4.38	0.003	0.062
Ruminococcus callidus	0 (0 - 0)	0 (0 - 0)	0 (0 - 0.0263)	0 (0 - 0)	0.00	0.036	0.231
Clostridium innocuum	0.0018 (0 - 0.0115)	0.0052 (0.0001 - 0.0387)	0.0035 (0 - 0.0098)	0.0019 (0 - 0.0112)	0.17	0.033	0.231
Clostridium spiroforme	0.0534 (0.0207 - 0.1253)	0.125 (0.0153 - 0.5476)	0.0181 (0.0068 - 0.0774)	0.0921 (0.0191 - 0.3824)	0.87	0.012	0.151
Turicibacter sanguinis	0.0005 (0 - 0.0109)	0 (0 - 0)	0 (0 - 0.0395)	0 (0 - 0)	0.00	0.019	0.201
Firmicutes bacterium CAG:110	0.0048 (0 - 0.4866)	0 (0 - 0.0011)	0.1175 (0.0124 - 0.3326)	0 (0 - 0.0281)	-2.54	0.025	0.224
Veillonella atypica	0 (0 - 0.017)	0 (0 - 0)	0 (0 - 0)	0 (0 - 0)	0.00	0.036	0.231
Veillonella dispar	0 (0 - 0.0082)	0 (0 - 0)	0 (0 - 0)	0 (0 - 0)	0.00	0.035	0.231
Veillonella parvula	0 (0 - 0.0461)	0 (0 - 0)	0 (0 - 0.0046)	0 (0 - 0)	0.00	0.014	0.168

Bilophila wadsworthia	0.0126 (0 - 0.0448)	0 (0 - 0)	0.0342 (0.0093 - 0.037)	0 (0 - 0)	-6.89	0.001	0.039
Akkermansia muciniphila	0.0542 (0 - 0.3363)	0 (0 - 0)	0.1716 (0 - 0.6631)	0 (0 - 0)	-7.99	0.003	0.06

*Log2 fold change, p-values and FDR-adjusted p-values are based on the combined changes for the erythromycin and azithromycin groups. Microbial species abundances are indicated as median and interquartile ranges.

Table S3. Microbial pathways that were significantly altered in healthy adults following exposure to erythromycin and/or azithromycin for 4 weeks.

Microbial pathways	Erythromycin (ERY)		Azithromycin (AZM)		Log2FC	p	FDR p
	Baseline	ABX	Baseline	ABX			
ANAEROFRUCAT-PWY:homolactic fermentation	5837.2 (4966.7, 6803.6)	4432.5 (3741.6, 4866.3)	5558.7 (4891.1, 6187.9)	3120.5 (2930, 3870.5)	-0.08	<0.001	0.001
COA-PWY:coenzyme A biosynthesis I (prokaryotic)	9569.1 (7999.6, 9895.7)	10457.9 (9529.4, 10822.8)	10145.5 (9534, 10416.6)	10441.8 (10331, 10873.4)	0.26	<0.001	0.003
FERMENTATION-PWY:mixed acid fermentation	2540.1 (1310.4, 3269.3)	765.1 (303.3, 1040.9)	2245 (1339.5, 2657.2)	450 (228.6, 520.8)	0	<0.001	<0.001
GLUCONEO-PWY:gluconeogenesis I	3669.1 (3101.6, 4538.4)	3069.8 (2358.1, 3403.9)	3947.7 (3436.6, 4366.2)	2186.9 (1921.6, 2687.1)	-4.59	<0.001	0.003
GLUCOSE1PMETAB-PWY:glucose and glucose-1-phosphate degradation	1290 (335.5, 2434.7)	0 (0, 41.7)	310.3 (127.4, 602.7)	0 (0, 25.3)	0	<0.001	<0.001
GLYCOCAT-PWY:glycogen degradation I	2418 (658.9, 3856.5)	0 (0, 83.2)	612.4 (252.7, 1168.6)	0 (0, 50.6)	0.28	<0.001	<0.001
GLYCOLYSIS:glycolysis I (from glucose-6-phosphate)	4947.4 (3260.5, 5631.8)	2668 (2429.6, 3074.4)	3710.5 (3383.1, 5144.6)	1690.1 (1498.2, 2401.2)	0	<0.001	0.002
P124-PWY:Bifidobacterium shunt	879.1 (434.2, 2425.6)	0 (0, 67.1)	1513.4 (988.5, 1649.1)	0 (0, 0)	0.06	<0.001	0.002
PWY-241:C4 photosynthetic carbon assimilation-cycle (NADP-ME type)	2204.2 (1082.4, 3350.1)	475.6 (198, 787.2)	1718.3 (894.6, 2324.4)	301.5 (102.7, 352.7)	-4.6	<0.001	<0.001
PWY-2723:trehalose degradation V	1314.1 (338.1, 2237.1)	0 (0, 41.7)	311.7 (127.7, 601.1)	0 (0, 25.4)	0.23	<0.001	<0.001
PWY-5384:sucrose degradation IV (sucrose-phosphorylase)	1426.4 (512.4, 2499.6)	141.3 (98, 194.6)	949.7 (560.8, 1625.6)	57.3 (46.5, 102.8)	0	<0.001	<0.001
PWY-5484:glycolysis II (from fructose-6-phosphate)	4397.4 (3032.5, 4907.3)	2371.7 (2127.2, 2598.2)	3406.3 (2726.6, 4432.5)	1540.5 (1336.8, 1965.6)	-0.56	<0.001	0.001
PWY-5913:partial TCA cycle (obligate autotrophs)	2412.8 (930.4, 3262)	415.2 (161, 686.8)	1494.6 (802.5, 2478.1)	253.1 (83.5, 292)	-1.44	<0.001	<0.001
PWY-622:starch biosynthesis	1132.1 (315.2, 3507.4)	0 (0, 0)	542.3 (180.8, 864.2)	0 (0, 0)	-0.41	<0.001	0.002
PWY-6527:stachyose degradation	6915.3 (6284.1, 7563.4)	8080.1 (7430.4, 9409.3)	7393.9 (6772.3, 8105.5)	9807.1 (9174.5, 10075.8)	-0.11	<0.001	0.002
PWY-6549:L-glutamine biosynthesis III	1993.6 (1297.2, 2164.8)	1018 (408.4, 1278.6)	1973.5 (1543.4, 2137.2)	567.5 (264.8, 646)	0	<0.001	0.001
PWY-6731:starch degradation III	1353.9 (411.7, 2161.6)	0 (0, 52)	372.1 (156.1, 709.3)	0 (0, 31.6)	0.12	<0.001	<0.001

PWY-7117:C4 photosynthetic carbon assimilation cycle (PEPCK type)	2502.6 (1198.8, 3316.1)	531.6 (231.3, 779.1)	1677.4 (949.5, 2586)	342.7 (120.7, 399.1)	0	<0.001	<0.001
PWY-7198:pyrimidine deoxyribonucleotides de novo biosynthesis IV	1523 (1107.6, 1812.3)	786 (649.1, 1175.6)	1616.9 (1236.2, 1987)	345.4 (222.1, 562.2)	0.14	<0.001	<0.001
PWY-7234:inosine-5'-phosphate biosynthesis III	3090.8 (1790.8, 3722.5)	1218.8 (476.5, 1793.8)	2399.6 (1081.4, 2916.2)	731.2 (459.7, 1081.7)	0.28	<0.001	0.001
PWY-7237:myo-, chiro- and scyllo-inositol degradation	6053.1 (5522.4, 6458.8)	7543.5 (6468.7, 7814.1)	6350.3 (5987.1, 7667.6)	9082.4 (8046, 9985.5)	-0.01	0.001	0.004
PWY-7328:superpathway of UDP-glucose-derived O-antigen building blocks biosynthesis	1420.6 (400.2, 2245.5)	19.5 (0, 64.7)	376.4 (157.2, 711.3)	0 (0, 31.3)	0	<0.001	<0.001
PWY-7357:thiamine phosphate formation from pyrithiamine and oxythiamine	7655 (6537.4, 8239)	8932.6 (7745, 9668.5)	8544.6 (7672.2, 8986.5)	10018.7 (9756.6, 10358.5)	0	<0.001	0.001
UDPNAGSYN-PWY:UDP-N-acetyl-D-glucosamine biosynthesis I	5213.9 (4351, 5936.8)	3288 (2789.3, 4198.4)	6046.2 (4754.9, 6583.7)	3465.2 (2917.6, 4055.7)	0	<0.001	0.003
1CMET2-PWY:folate transformations III (E. coli)	8726.6 (8300.5, 9727.9)	9870.1 (9222.5, 10162.4)	9474.7 (9195, 9783.1)	10300.7 (9763.5, 10968.6)	0	<0.001	0.002
HEXITOLDEGSUPER-PWY:superpathway of hexitol degradation (bacteria)	2314.7 (1947.1, 2680.1)	1801.8 (1530.5, 2238.6)	1935.8 (1596.8, 2357.5)	1417.2 (1033.4, 1546)	-0.21	0.003	0.012
PANTO-PWY:phosphopantothenate biosynthesis I	6776.6 (5772.7, 7882.8)	8033.9 (7813.8, 8659.4)	7151.1 (6474.2, 7662.2)	8812 (8396.3, 9466.1)	0.24	<0.001	0.001
PANTOSYN-PWY:superpathway of coenzyme A biosynthesis I (bacteria)	7498.6 (6053.7, 8474.7)	8627 (8501.9, 9154.6)	7905 (7573.8, 8517.5)	9300.1 (9076.2, 9993.3)	-0.88	<0.001	0.001
PWY-1042:glycolysis IV	12830.8 (11800.9, 13661.8)	14774.5 (13796.8, 15556.4)	13392.3 (12527.3, 13750.1)	14910.2 (14445.8, 15828.8)	-0.03	<0.001	<0.001
PWY-4041:gamma;-glutamyl cycle	2440.1 (1828.3, 2667.1)	1327.1 (1134.9, 1566.3)	2077.1 (1849.8, 2462.4)	1212.5 (1010.7, 1422.4)	0.11	<0.001	0.001
PWY-5667:CDP-diacylglycerol biosynthesis I	9712.5 (8687, 10328.5)	10846.9 (10014.7, 11377.8)	10332.7 (9764.5, 10501)	11063.6 (10850.6, 11468.2)	0	<0.001	0.001
PWY-5690:TCA cycle II (plants and fungi)	1436.7 (1031.4, 1958.2)	924.5 (497.9, 1048.2)	999.8 (858.6, 1323.2)	498.3 (352.3, 715.5)	0	<0.001	0.001
PWY-6168:flavin biosynthesis III (fungi)	7106 (6047.6, 7854.8)	8382.5 (7545.7, 8817.9)	7628.7 (6745.9, 7866.4)	9334.5 (8934.8, 9591)	0	<0.001	0.001
PWY-6317:D-galactose degradation I (Leloir pathway)	6970.7 (6141.8, 7681.7)	8554.7 (7890.8, 8804.9)	7519.3 (6929.4, 7791.8)	9340.4 (9241.5, 9606.4)	0	<0.001	<0.001

PWY-6901:superpathway of glucose and xylose degradation	2869.6 (2407.8, 3550.2)	1461.7 (1351.6, 1596)	2332.9 (1972.4, 2997)	1180.1 (958.6, 1495.7)	0.17	<0.001	<0.001
PWY0-1296:purine ribonucleosides degradation	9219.3 (7985.3, 9856.4)	11383.2 (10329.4, 12197.9)	10289 (9194.9, 10769)	11753.4 (11515.6, 12477.8)	0.35	<0.001	0.001
PWY0-1319:CDP-diacylglycerol biosynthesis II	9712.5 (8687, 10328.5)	10846.9 (10014.7, 11377.8)	10332.7 (9764.5, 10501)	11063.6 (10850.6, 11468.2)	0	<0.001	0.001
PWY66-399:gluconeogenesis III	1779.1 (1311.5, 2051.2)	1329 (1097.7, 1844.5)	1836.5 (1699.8, 2225.7)	1178.5 (864.9, 1309.5)	0.18	0.001	0.004
PWY66-422	6970.7 (6141.8, 7681.7)	8348.3 (7682.5, 8804.2)	7519.3 (6929.4, 7791.8)	9340.4 (9241.5, 9606.4)	-2.18	<0.001	0.001
PYRIDNUCSYN-PWY:NAD de novo biosynthesis I (from aspartate)	6875.8 (5762.8, 7136.1)	7289.8 (6957, 7860.6)	6494.1 (5447.8, 7252.4)	7395.2 (7151, 7735.3)	-0.44	<0.001	0.001
TCA:TCA cycle I (prokaryotic)	1982.1 (1429.3, 2501)	1272 (652.2, 1457.5)	1325.3 (1227, 1701.2)	730.7 (517.7, 1034.9)	0.13	<0.001	0.001
FASYN-INITIAL-PWY:superpathway of fatty acid biosynthesis initiation (E. coli)	8773.1 (7956.3, 9385.6)	10353.5 (8914.3, 10532.2)	9175.7 (7823.7, 9593.5)	10041.3 (9999.2, 10215.3)	-2.23	<0.001	0.003
GLYCOLYSIS-E-D:superpathway of glycolysis and the Entner-Doudoroff pathway	1968.5 (1351, 2157.8)	1241.1 (1068.9, 1354.4)	1525.9 (1445, 1886.4)	780.9 (429.2, 974.6)	0	<0.001	0.001
OANTIGEN-PWY:O-antigen building blocks biosynthesis (E. coli)	6481.7 (5724.3, 7226.3)	5064.5 (4444.2, 5648.7)	7249.4 (6488, 7827.3)	5197.5 (4581.3, 5882.2)	0	0.002	0.007
PWY-6151:S-adenosyl-L-methionine salvage I	10984.4 (9653.9, 11212.1)	11503.6 (10983.1, 11800.8)	11676.5 (10969.8, 11830.3)	12070.2 (11757.5, 12785)	0.15	0.001	0.004
PWY0-1479:tRNA processing	1864.3 (1716, 2166.1)	875.5 (702.3, 1387.9)	1283.7 (1077.3, 1579.5)	618.9 (505.9, 715.6)	0	<0.001	<0.001
RIBOSYN2-PWY:flavin biosynthesis I (bacteria and plants)	7603.7 (6417, 8457.4)	8828.3 (7991.3, 9269.6)	8000.3 (7166.7, 8338.6)	9731.7 (9308.1, 9970.2)	0.06	<0.001	0.002
THISYNARA-PWY:superpathway of thiamine diphosphate biosynthesis III (eukaryotes)	4973.9 (4579, 5346.3)	5736.2 (4670.2, 6907.4)	4830.5 (4422.6, 6097.2)	6985.5 (5900.5, 7707.6)	0	0.001	0.004
TRPSYN-PWY:L-tryptophan-biosynthesis	7014.1 (6434.3, 7457.6)	7622.7 (6329.2, 8887.8)	7627.1 (6674.6, 8143.2)	8878.7 (8495.7, 9794.9)	0	0.002	0.007
COMPLETE-ARO-PWY:superpathway of aromatic amino acid biosynthesis	11464 (10514.1, 11683.8)	12087 (11411.9, 12573.3)	12014.6 (11579, 12739.6)	12993 (12542.5, 13406)	0	<0.001	0.002
P4-PWY:superpathway of L-lysine, L-threonine and L-methionine biosynthesis I	2531.6 (1390, 3764.2)	652.9 (403.5, 1397.2)	1909 (1441.7, 3087.3)	694.7 (597.9, 923.6)	-0.63	<0.001	0.002

PWY-4242	10020.8 (8225.5, 10273.1)	10994.9 (9884.7, 11338.2)	10503.5 (9939.7, 10910.5)	10947 (10655.3, 11399.9)	0	0.001	0.004
PWY0-781:aspartate superpathway	2372.3 (1455.9, 3395.6)	688.5 (427.7, 1453.7)	1995.2 (1514.6, 2825)	735 (630.4, 975.9)	-0.41	<0.001	0.002
CALVIN-PWY:Calvin Benson Bassham cycle	9576.8 (8589.4, 10552.1)	10971.7 (10559.4, 11512.9)	10441.8 (9854.9, 10879.4)	11694.9 (11480.4, 12068.4)	0	<0.001	0.001
COBALSYN-PWY:superpathway of adenosylcobalamin salvage from cobinamide I	5094.8 (4341.2, 5710.5)	6172.7 (5381.3, 6996)	5750.2 (5226.5, 6181.4)	7313.7 (6491.2, 7576.5)	-0.18	<0.001	0.002
PWY-6163:chorismate biosynthesis from 3-dehydroquinate	12413.4 (11405.7, 12840.9)	13610.9 (13078.5, 14209.9)	12867.9 (12489.7, 14100.2)	14228.7 (13802.3, 14805.3)	0	<0.001	0.002
PWY-6969:TCA cycle V (2-oxoglutarate-synthase)	1227.1 (1033.9, 1647.6)	1016.5 (723.9, 1235)	872.8 (670.4, 1403.1)	432.5 (282.4, 810)	0.29	0.002	0.007
PWY-7388:octanoyl-[acyl-carrier protein] biosynthesis (mitochondria, yeast)	1902.2 (1077.7, 3188.4)	351 (153.3, 958.7)	1755.4 (508.9, 2244.6)	339.3 (133.9, 761.8)	-0.06	<0.001	0.003
PWY-7209:superpathway of pyrimidine ribonucleosides degradation	537.2 (106.9, 861.5)	0 (0, 0)	441.4 (68.5, 724.1)	0 (0, 0)	0.06	<0.001	0.002
ARO-PWY:chorismate biosynthesis I	12228.8 (11277.1, 12534.7)	13276.3 (12691.1, 13610.1)	12726.2 (12412.2, 13578.8)	13672.5 (13413, 14293)	0.24	0.001	0.004
NONOXIPENT-PWY:pentose phosphate pathway (non-oxidative branch) I	7788.3 (7061.3, 8649.8)	8863.2 (8424.3, 10225.1)	7846.8 (7590.7, 8955.2)	9740.3 (8760.7, 9997.3)	-0.47	0.001	0.004
PWY-6282 (palmitoleate-biosynthesis-I (from (5Z (dodec-5-enoate-	962 (844.3, 2222.1)	318.3 (150.8, 789.7)	1197.6 (433.3, 1612.5)	269.8 (153.9, 648)	-0.78	0.001	0.004
PWY-5918:superpathway of heme b biosynthesis from glutamate	106.9 (58, 452.4)	75.4 (47, 134.1)	89 (56.4, 147.5)	0 (0, 33.8)	-0.92	0.021	0.065
PWY-7210:pyrimidine deoxyribonucleotides biosynthesis from CTP	833.6 (235.8, 984)	326 (95, 712)	30.5 (0, 41.7)	176.7 (12.3, 428)	-0.1	0.737	0.788
PWY0-1261:anhydromuropeptides recycling I	3826.3 (2353, 4137.7)	2661.3 (1695.4, 3205.2)	2640.8 (2044.1, 3241.6)	1483.7 (805.5, 2044.8)	0	0.001	0.004
BIOTIN-BIOSYNTHESIS-PWY:biotin biosynthesis I	1182.2 (998.2, 2105.5)	412.9 (220.9, 1001.7)	1472.1 (513.5, 1796.6)	383.5 (209.5, 808.8)	0.06	0.001	0.004
GLYCOGENSYNTH-PWY:glycogen biosynthesis I (from ADP-D-Glucose)	9110.7 (8832.3, 10362.2)	10956.6 (10008.3, 12489.4)	10376.9 (9824.5, 10604.6)	12436.2 (10967.2, 12982.1)	0	0.001	0.004
HSERMETANA-PWY:L-methionine biosynthesis III	6622.2 (6094.7, 7655.6)	8381.1 (6922.5, 9372.2)	7346.2 (7223.9, 7724.5)	8213.8 (7771.1, 8794.2)	0.03	0.002	0.008

NONMEVIPP-PWY:methylerythritol phosphate pathway I	11234.6 (10873.2, 11560.2)	11955.9 (11428, 12202.7)	11751.6 (11651.5, 12034.8)	12230.4 (11750.7, 12896.1)	0.24	0.005	0.018
P42-PWY:incomplete reductive TCA cycle	482.2 (401.3, 763.5)	395.1 (283.6, 911)	475.3 (292.6, 886.2)	219.1 (154.9, 420.2)	-0.39	0.04	0.103
P441-PWY:superpathway of N-acetylneuraminate degradation	1959.7 (1779.5, 2147.3)	1542.9 (1343, 1872.4)	1600.6 (1002.9, 1779.7)	1082.3 (865.5, 1279.6)	0.32	0.008	0.03
PENTOSE-P-PWY:pentose phosphate pathway	2914.4 (2117.1, 3267.8)	1578.5 (872.7, 1699.3)	1768.5 (1391.7, 2509.9)	1182.9 (685.9, 1362.8)	0.03	<0.001	0.003
PWY-5100:pyruvate fermentation to acetate and lactate II	6551 (5820.6, 7250.6)	8154.7 (7475.3, 9494.4)	7745.8 (6972.3, 8329)	8418.5 (8117.9, 9263.2)	0	<0.001	0.002
PWY-6270:isoprene biosynthesis I	7917.2 (7523.6, 8366)	8982.1 (8390.9, 9724.8)	8154.4 (7879.9, 8320.2)	9278.3 (8384.4, 9714.1)	0.06	<0.001	0.003
PWY-6737 :starch degradation V	14334.5 (13540.4, 15454.6)	15797 (14855.6, 16692.5)	14555.3 (14063.5, 15222.9)	15862.8 (15541.6, 16550.4)	0	<0.001	0.001
PWY-6897:thiamine-diphosphate-salvage-II	5175.2 (4435.9, 5659.6)	5773.9 (5021.7, 7067.7)	5165.9 (4693.3, 6341.4)	6630.9 (5915.3, 7705.2)	-0.9	0.002	0.008
PWY-7560:methylerythritol phosphate pathway II	7759.1 (7432.2, 8239)	9051.9 (8298, 9641.1)	8015.1 (7733.4, 8140.2)	9295.1 (8388, 9660.1)	0	<0.001	0.003
PWY-7664:oleate biosynthesis IV (anaerobic)	1084.3 (943.8, 2429.5)	328.5 (137.3, 861.7)	1334.5 (442.2, 1793.6)	308.1 (129.3, 692.7)	0	0.001	0.004
PWY0-862:(5Z)-dodecenoate biosynthesis I	995.8 (871.5, 2352.5)	289 (119, 774.2)	1265.2 (397.7, 1688.2)	273.5 (112.3, 617.6)	0	0.001	0.004
PWY-5083:NAD(P)/NADPH interconversion	26.1 (0, 80)	12.6 (0, 95)	23.7 (0, 89.3)	0 (0, 0)	0	0.052	0.117
PWY0-1415:superpathway of heme b biosynthesis from uroporphyrinogen-III	46.3 (31.3, 252.3)	19.3 (0, 78.3)	33.5 (0, 74.4)	0 (0, 0)	-0.78	0.036	0.096
DAPLYSINESYN-PWY:L-lysine biosynthesis I	3331.7 (2379.9, 4439.4)	1193.3 (840.2, 2085.2)	2684.1 (1704.1, 3978.2)	1020.7 (725.3, 2166.1)	0	0.001	0.003
FASYN-ELONG-PWY:fatty acid elongation	1163.3 (991, 2492.8)	366 (155.2, 941.7)	1395.8 (483.6, 1864)	340.5 (145.9, 762.1)	0.19	0.001	0.004
PWY-5005:biotin biosynthesis II	282.1 (157.4, 579.5)	329.8 (126.1, 649.4)	320.3 (222.9, 742)	361.6 (96.5, 556.1)	-0.25	0.344	0.434
PWY-6519:8-amino-7-oxononanoate biosynthesis I	996.9 (878.6, 2017.7)	335 (176.4, 855.9)	1313.5 (422.3, 1583.2)	312.3 (166.7, 679.2)	0.36	0.001	0.004
GALACTARDEG-PWY:D-galactarate degradation I	90.2 (16.1, 239.3)	84.1 (42.2, 103.7)	146.2 (14.5, 201)	30.8 (2.9, 108.1)	-0.32	0.107	0.177
GLUCARDEG-PWY:D-glucarate degradation I	81.7 (9.7, 103.7)	76.5 (21.1, 97.7)	75.1 (4.6, 126.3)	12.6 (0, 30.5)	-0.21	0.151	0.225

GLUCARGALACTSUPER-PWY:superpathway of D-glucarate and D-galactarate degradation	90.2 (16.1, 239.3)	84.1 (42.2, 103.7)	146.2 (14.5, 201)	30.8 (2.9, 108.1)	-0.61	0.107	0.177
PWY-5173	105.5 (54.5, 665.4)	30.7 (15.8, 56.9)	41.1 (10.2, 112.5)	15.9 (0, 37)	0	0.012	0.04
PWY-5464:superpathway of cytosolic glycolysis (plants), pyruvate dehydrogenase and TCA cycle	172.4 (37.3, 263.2)	97.5 (64.8, 165.1)	101.4 (32.2, 190.5)	28.3 (0, 72)	-0.9	0.139	0.214
ASPASN-PWY:superpathway of L-aspartate and L-asparagine biosynthesis	3576 (3469, 3932.7)	4442.8 (4145.9, 4671.3)	3532.5 (3376.8, 3825.9)	4491.4 (3926.2, 4968.1)	0	0.001	0.004
DTDPRHAMSYN-PWY:dTDP-β-L-rhamnose biosynthesis	11317.9 (10391.1, 11911.9)	13171 (12797.5, 13704.7)	11148.3 (10650.6, 12586.9)	12426.7 (12059.1, 13008.2)	0	<0.001	0.003
GLUTORN-PWY:L-ornithine biosynthesis I	9112.3 (8393.7, 10268.2)	10027.8 (9118.8, 10409.7)	10485.8 (9375.6, 10841.7)	10901.2 (10771.2, 11053.8)	-5.19	0.012	0.041
X-GOLPDLCAT-PWY:superpathway of glycerol degradation to 1,3-propanediol	804.1 (768, 853)	1260.5 (1063.6, 1405.7)	678.3 (617.9, 808.4)	1206 (736.9, 1371.8)	0	0.002	0.009
PWY0-1241:ADP-L-glycero-β-D-manno-heptose biosynthesis	378.8 (266.5, 867.3)	230.6 (179.2, 319.2)	220.1 (143.1, 456.2)	98.2 (2.2, 133.9)	-0.48	0.001	0.004
PYRIDNUCSAL-PWY:NAD salvage pathway I (PNC VI cycle)	1885.1 (514.2, 3394.8)	354 (89.2, 851.4)	1429.6 (343.1, 3043.4)	188.8 (114, 435)	0.17	0.002	0.007
PWY-7254:TCA cycle VII (acetate-producers)	90.5 (8.5, 824.3)	0 (0, 23.3)	8.3 (0, 162.2)	0 (0, 0)	0	0.003	0.013
P164-PWY:purine nucleobases degradation I (anaerobic)	1625.6 (1310.4, 1929.8)	2001.4 (1762.2, 2472)	1946.8 (1636.2, 2087.9)	2643 (2089.2, 2859.5)	0.04	<0.001	0.003
PWY-6284:superpathway of unsaturated fatty acids biosynthesis (E. coli)	489.9 (386.7, 823)	288.5 (135.3, 369)	531.8 (370.9, 742.8)	183.2 (76.5, 456.8)	0	0.001	0.004
HEME-BIOSYNTHESIS-II:heme b biosynthesis I (aerobic)	113.2 (40.7, 452.7)	46.1 (26.4, 121.1)	78.1 (40.1, 94)	8.5 (0, 38.7)	-0.65	0.003	0.01
METH-ACETATE-PWY:methanogenesis from acetate	2629.2 (2095, 2854.6)	3446 (2865.5, 4015.7)	2739.7 (2105.6, 3245.5)	3834 (2991, 4285.1)	0	0.001	0.004
PWY-5121:superpathway of geranylgeranyl diphosphate biosynthesis II (via MEP)	2135.4 (1809.1, 3264.6)	1544.8 (922, 2668.9)	2347.7 (2023.7, 2656)	1746.9 (1108.3, 2530.6)	0	<0.001	0.003
PWY-7115:C4 photosynthetic carbon assimilation cycle, NAD-ME type	1533.8 (1263, 2125.7)	942.4 (783.7, 1060.7)	1234.5 (1022.8, 2013.6)	898.1 (698.5, 962.9)	-2.44	0.001	0.004
REDCITCYC:TCA cycle VI (Helicobacter)	89.9 (38.5, 194)	0 (0, 53.2)	35.4 (4.6, 168.1)	19.9 (0, 36.7)	0.03	0.005	0.019

PWY-6121:5-aminoimidazole ribonucleotide biosynthesis I	10078 (9633.7, 10326.6)	10764.1 (10175.7, 11801.4)	11036.2 (10499.2, 11470.6)	11471.9 (11196.6, 11721.4)	0.06	0.008	0.03
PWY-6609:adenine and adenosine salvage III	11502.7 (9670.9, 12019.8)	13118.7 (12497.3, 14188.5)	12285.4 (11739.7, 13139.1)	13107.1 (12667.8, 13540.2)	0	0.001	0.003
PWY-5971:palmitate biosynthesis (type II fatty acid synthase)	1220.4 (956.8, 2168.6)	425.6 (183.9, 950.2)	1012.1 (453.1, 1643.1)	375.5 (174.8, 679.7)	-0.56	0.004	0.016
PWY-5989:stearate biosynthesis II (bacteria and plants)	985.3 (861.1, 2239.8)	766.6 (242.7, 1095.7)	1234.9 (554, 1638.5)	667.8 (367.4, 974.2)	0	0.014	0.045
PWY-724:superpathway of L-lysine, L-threonine and L-methionine biosynthesis II	8725.8 (8445.8, 8862.5)	9058.3 (8716.6, 9149.9)	8984.3 (8737.7, 9206.3)	9209.7 (8915.6, 9442)	-1.04	0.048	0.113
SER-GLYSYN-PWY:superpathway of L-serine and glycine biosynthesis I	6478.6 (5872.4, 6783.4)	7050.5 (6716.9, 7581.1)	6911.8 (6714.7, 7098.2)	7662.9 (6386.2, 7963.4)	-0.86	0.058	0.12
PWY-7219:adenosine ribonucleotides de novo biosynthesis	15638.6 (14912.5, 16817)	17283.4 (16468, 17787)	16773.3 (15866.5, 17655)	16772.6 (16249.9, 17671.5)	-0.14	0.024	0.07
PWY0-1298:superpathway of pyrimidine deoxyribonucleosides degradation	405.3 (284.6, 757.3)	255.2 (181, 344.9)	373.9 (270.8, 555.2)	359.4 (155, 603.3)	0.14	0.044	0.11
PWY-6700:queuosine biosynthesis I (de novo)	10080.7 (9478.6, 11298.7)	10977.4 (10299.5, 11788.1)	11091.7 (10455.6, 11948.4)	11452.2 (10787, 11597.5)	0.21	0.083	0.145
P621-PWY:nylon-6-oligomer-degradation	29.9 (4.1, 45.3)	0 (0, 10)	21 (0, 33.8)	9.2 (0, 37.2)	0.31	0.038	0.099
PWY-6122:5-aminoimidazole ribonucleotide biosynthesis II	10686.8 (9866.2, 11680.2)	11785.3 (10707.7, 12553.1)	12117.3 (11353.9, 12394)	11858.8 (11411.5, 12437.8)	0	0.083	0.145
PWY-6277:superpathway of 5-aminoimidazole ribonucleotide biosynthesis	10686.8 (9866.2, 11680.2)	11785.3 (10707.7, 12553.1)	12117.3 (11353.9, 12394)	11858.8 (11411.5, 12437.8)	-1.39	0.083	0.145
TRNA-CHARGING-PWY:tRNA charging	11652.8 (10760.4, 12091.2)	12167 (11327.6, 13224.3)	12182 (11355.2, 12891.5)	11930.6 (11611.5, 12281.2)	0.25	0.33	0.419

*Log2 fold change, p-values and FDR-adjusted p-values are based on the combined changes for the erythromycin and azithromycin groups. Pathway abundance are indicated as the median and interquartile ranges.

Table S4. Total faecal bacterial load in conventional and germ mice model studies at baseline and at day 90.

**Erythromycin mice model in conventionally raised mice**			
	**Baseline**	**ABX**	**p**
**Control**	1.3×10^7 ($3.2 \times 10^6 – 1.5 \times 10^7$)	2.8×10^7 ($2.0 \times 10^7 – 3.5 \times 10^7$)	0.002
**Erythromycin**	5.7×10^6 ($1.5 \times 10^6 – 1.2 \times 10^7$)	1.3×10^7 ($4.0 \times 10^6 – 1.8 \times 10^7$)	0.451
**Erythromycin mice model in germ-free mice**			
	**Baseline**	**Day 90**	**p**
**Germ-free mice (GF)**	4.6 (2.6 – 8.0)	3.6 (2.9 – 4.0)	1.0
**Germ-free mice receiving erythromycin (GF-ERY)**	2.4 (2.4 - 3.4)	3.8 (2.6 – 3.9)	1.0
**Germ-free mice colonised with control microbiota (Conv-Con)**	3.1 (2.4 – 4.2)	1.0×10^7 ($8.5 \times 10^6 – 1.3 \times 10^7$)	<0.001
**Germ-free mice colonised with erythromycin-associated microbiota (Conv-ERY)**	4.4 (3.1 – 7.7)	5.6×10^7 ($5.2 \times 10^6 – 6.2 \times 10^7$)	<0.001

Data represents the median and interquartile ranges of faecal samples (copies of 16S rRNA gene/µL). ABX refers to day 90 following treatment with erythromycin ethylsuccinate (20mg/kg) or plain drinking water for control mice. The p-values were determined based on a linear mixed effects model with the cage effect modelled as random effect.

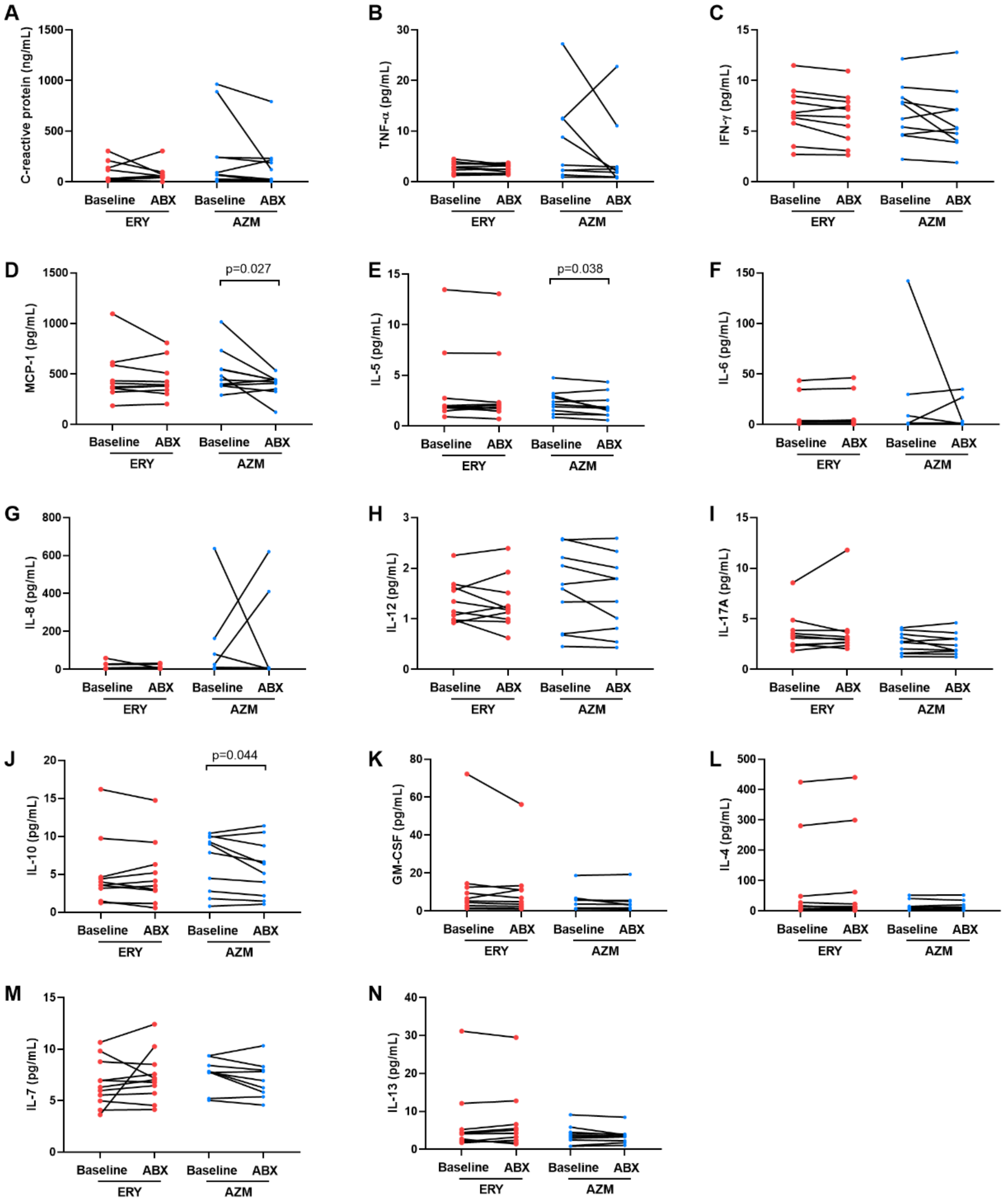

Figure S1. Host serum levels of inflammatory markers at baseline and at the end of 4-weeks antibiotic treatment (ABX) for the erythromycin (ERY) or azithromycin (AZM) groups. The levels of C-reactive protein (CRP), as wel as cytokines and chemokines including tumour necrosis factor (TNF)-α, interferon gamma (IFN-γ), monocyte chemoattractant protein 1 (MCP-1), IL-5, IL-6, IL-8, IL-12, IL-17A, IL-10, granulocyte-macrophage colony stimulating factor (GM-CSF), IL-4, IL-7 and IL-13 were determined using single or multiplex immunoassays as indicated in the Methods. Other immune-related markers including the hepatocyte growth factor (HGF), FGF-2, IL-1β IL-9, as well as the proinflammatory molecule lipopolysaccharide (LPS) were also measured but were detected in less than 60% of samples in either group and therefore, were not analysed further. Pairwise comparisons were performed using a linear mixed model (*lme4* version 1.1-23 and *lmerTest* 3.1-1 package in R), and multiple comparison correction of the p-value was performed using the false discovery rate (FDR) method. Statistical significance based on FDR $p < 0.05$ is shown.

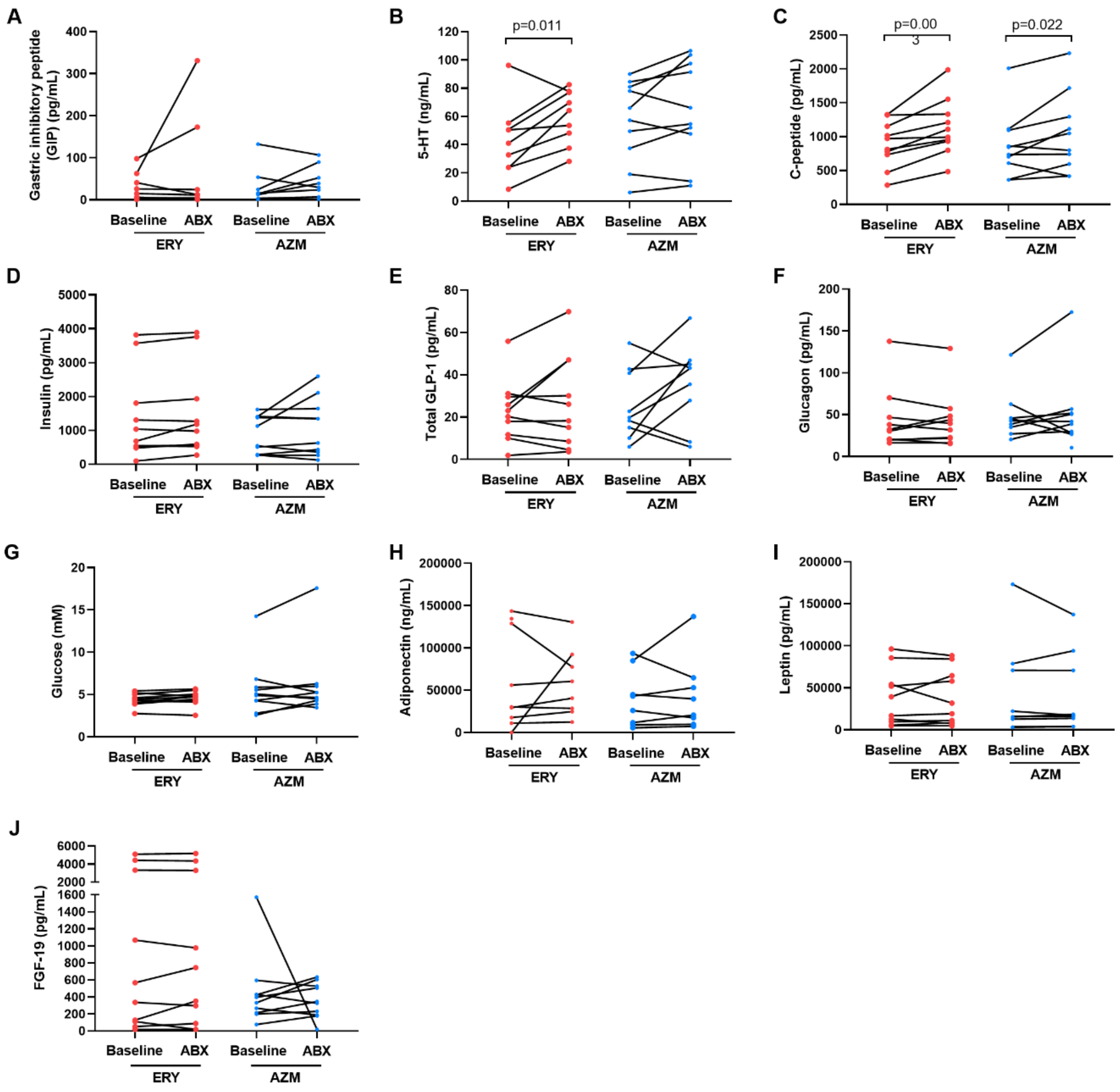

Figure S2. Host serum levels of metabolic markers at baseline and at the end of 4-weels antibiotic treatment (ABX) for the erythromycin (ERY) or azithromycin (AZM) groups. The levels of biomarkers including gastric inhibitory peptide, 5-HT (serotonin), C-peptide, insulin, total glucagon-like peptide 1 (GLP-1), glucagon, glucose, adiponectin, leptin, and FGF-19 were determined using single or multiplex immunoassays as indicated in the Methods. Serum levels of peptide YY (PYY) were also measured but were detected in less than 60% of samples in either group and therefore, were not analysed further. Pairwise comparisons were performed using a linear mixed model (*lme4* version 1.1-23 and *lmerTest* 3.1-1 package in R), and multiple comparison correction of the p-value was performed using the false discovery rate (FDR) method. Statistical significance based on FDR $p < 0.05$ is shown.

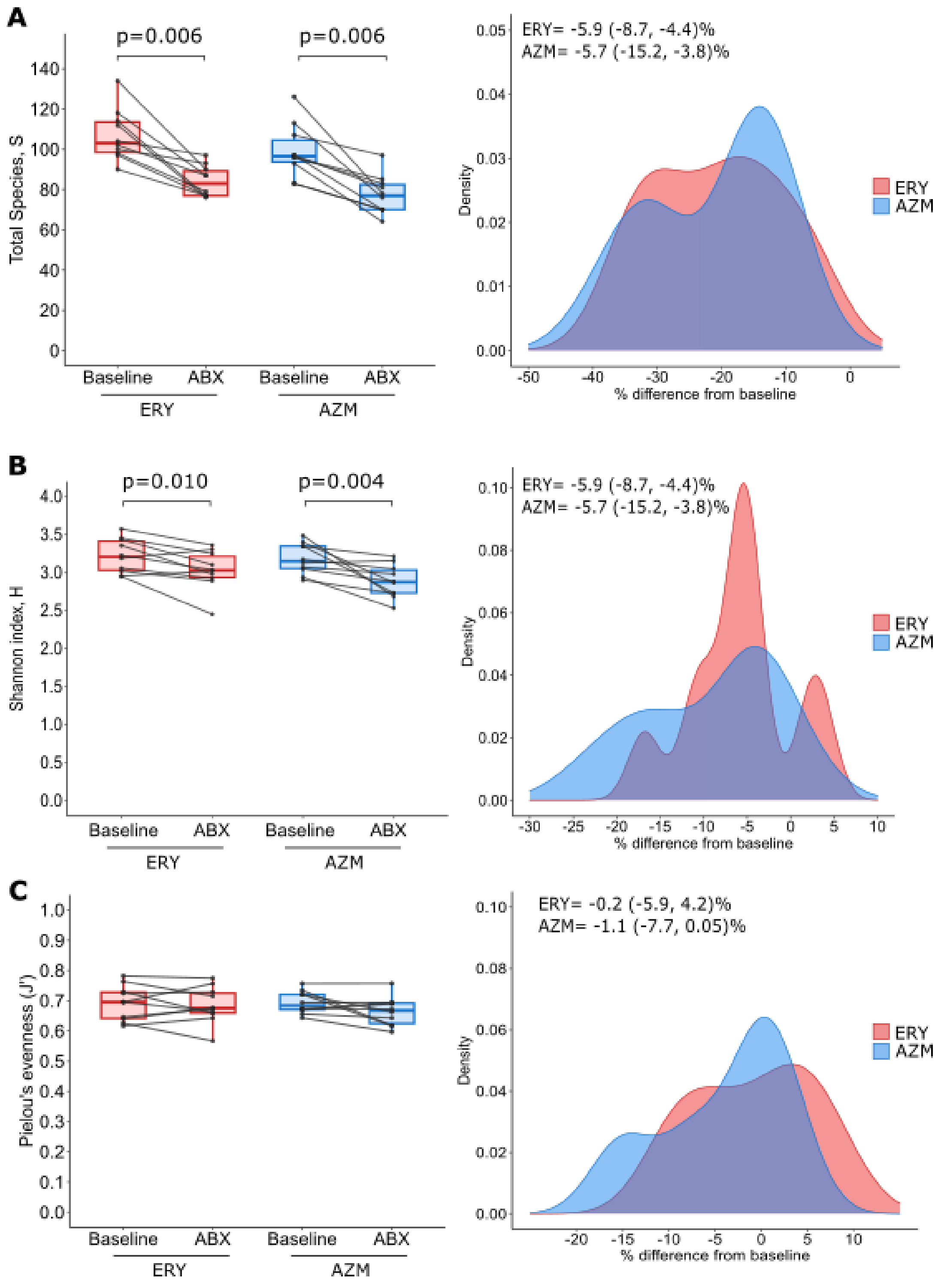

Figure S3. Alpha diversity measures of (A) species richness (Total species, S), (B) diversity (Shannon's diversity index (H') evenness (Pielou's J) at baseline and at the end of the 4 weeks of macrolide treatment (ABX) with erythromycin (ERY) or azithromycin (AZM). Statistical comparisons were performed using the Wilcoxon test at a significance level of $p < 0.05$. The corresponding density plot for each alpha diversity measure represents the distribution of the % of change after 4 weeks of treatment compared to their baseline values. The median and interquartile ranges of the % change is indicated.

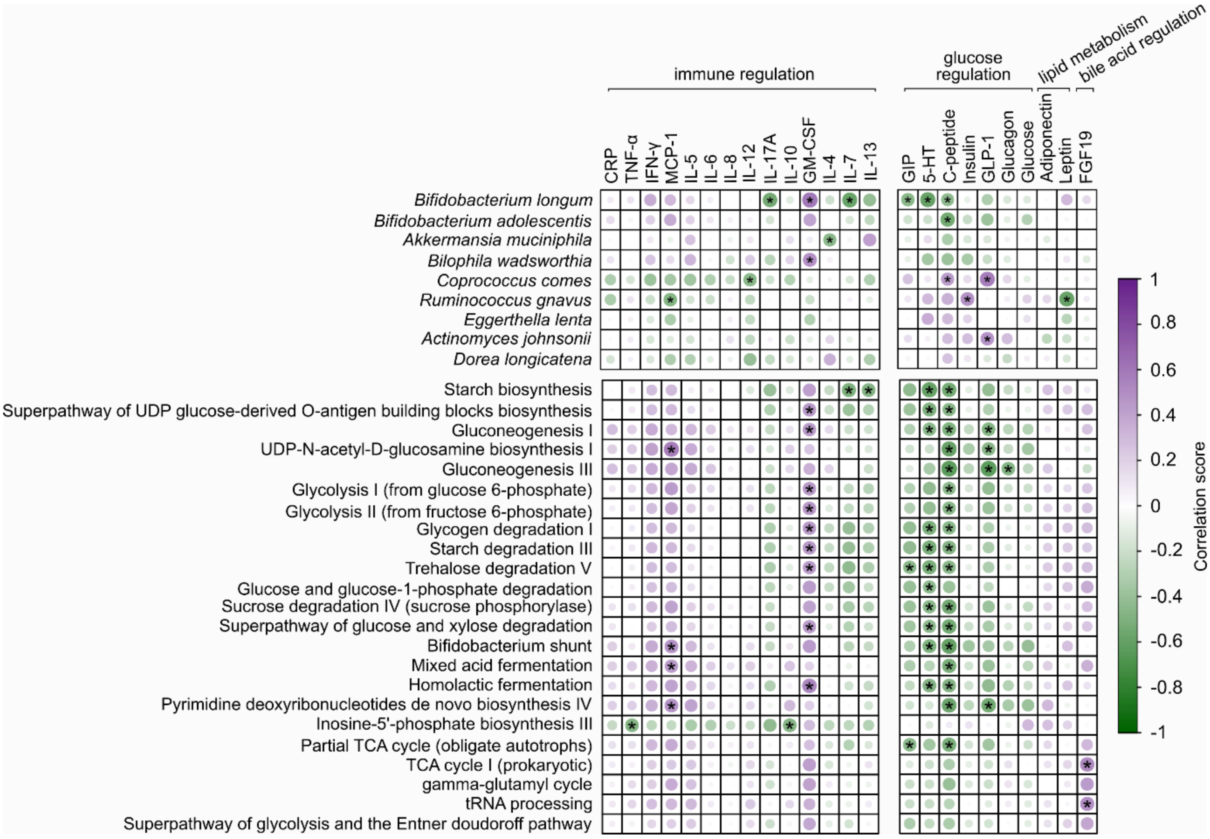

Figure S4. Correlation analysis between significantly altered microbial taxa or functional pathways and host biomarkers associated with immune and metabolic regulation. Correlation among the paired measures was assessed using the repeated measures correlation R package *rmcorr* (v0.5.4). Correlations with a significance level of $p<0.05$ are indicated with an asterisk (*).

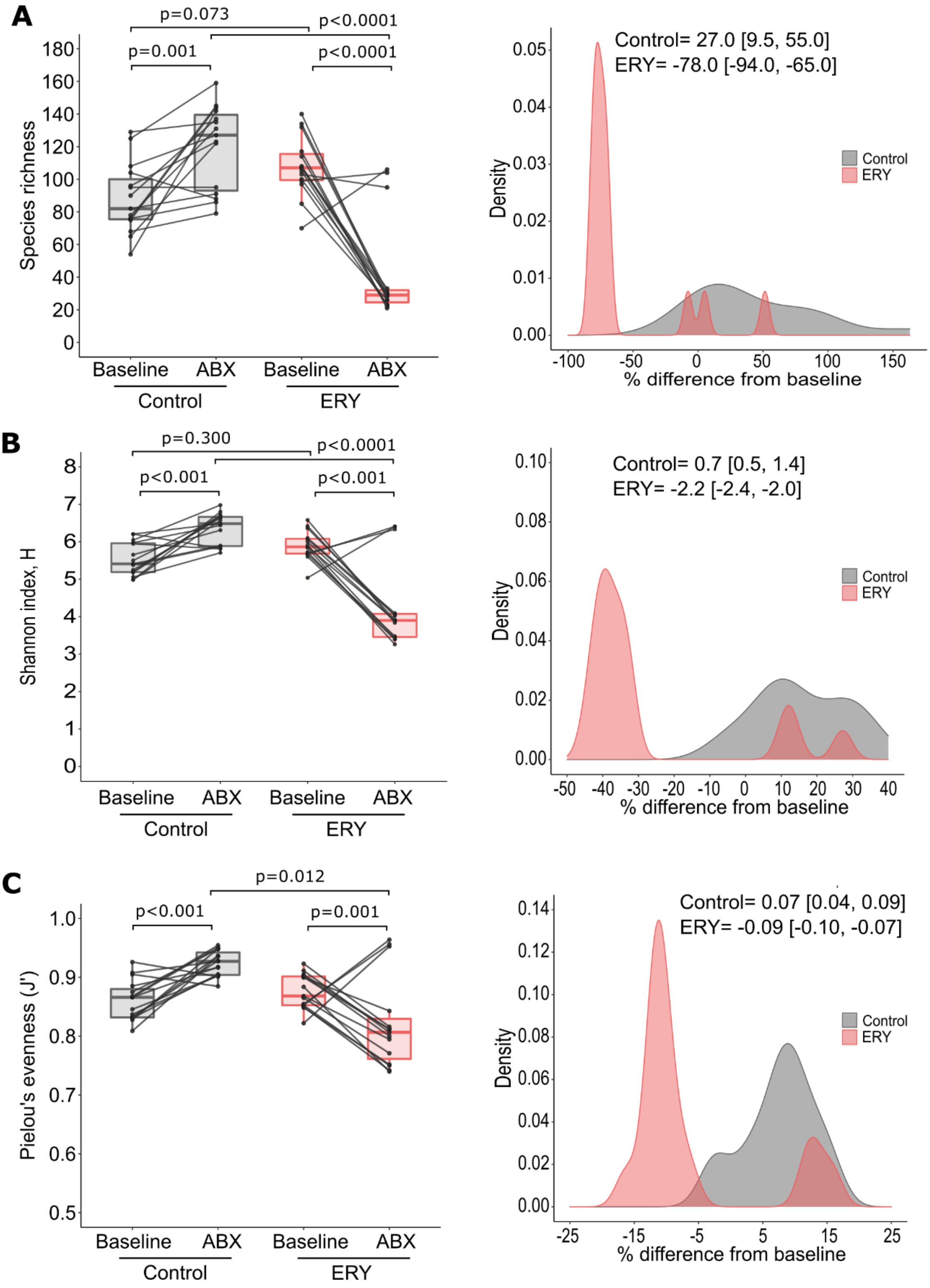

Figure S5. Faecal microbial alpha diversity based on (A) microbial richness (observed species), (B) diversity (Shannon's diversity index, H) and (C) evenness (Pielou's J). Significance of the changes between paired samples were determined using the Wilcoxon test at a level of $p < 0.05$. The corresponding density plot for each alpha diversity measure represented the % of change at day 90 compared to the baseline values. The median and interquartile ranges (first and third quartile, respectively, indicated in brackets) of the % change is indicated.

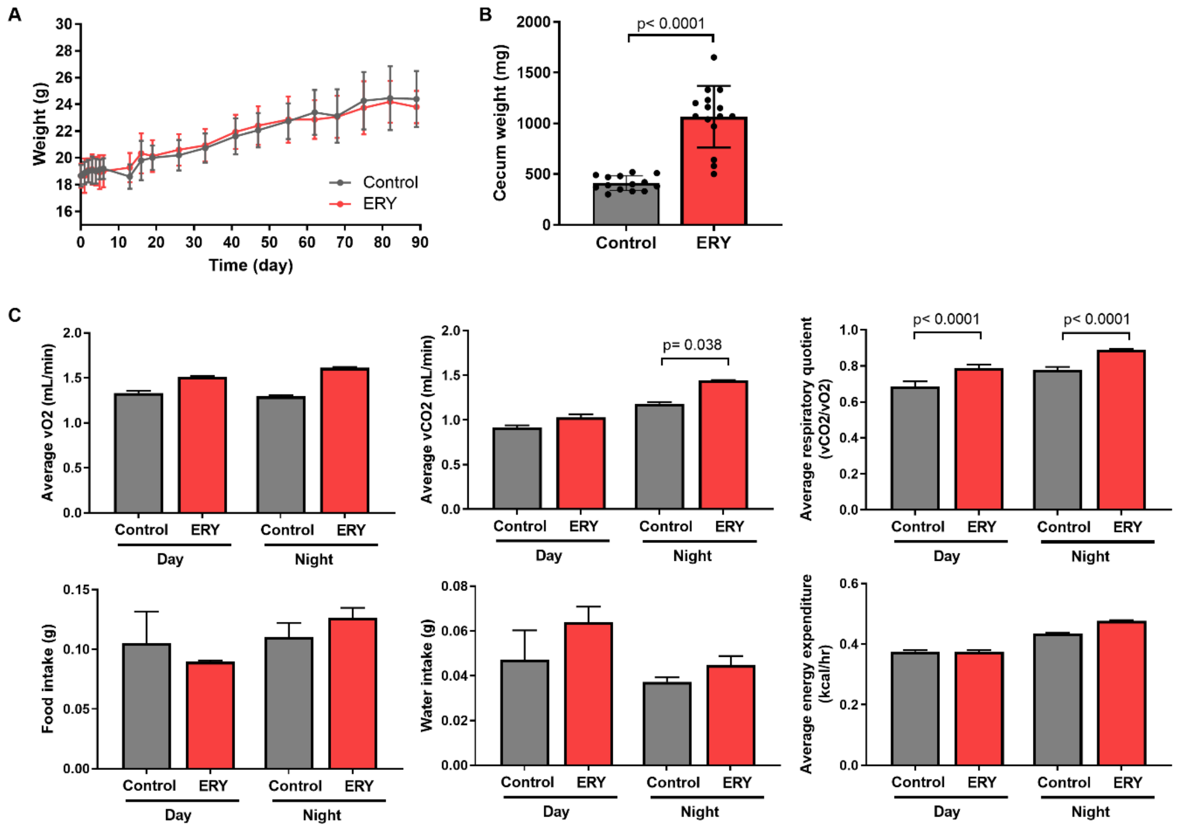

Figure S6. Physiological assessment of conventional mice exposed to erythromycin in drinking water (ERY) or control mice based on body weight over the 90 days study, and cecum weight at the end of the 90-day treatment (n=15 per group). (C) Metabolic output of individual erythromycin-treated (ERY) and control mice (n=3/group) were measured at the end of the 90-day treatment. Metabolic assessments were based on the average of the volume of CO_2 exhaled (vCO_2), volume of O_2 inhaled (vO_2), the overall respiratory quotient (calculated based on vCO_2/vO_2), food intake, water intake and the average energy expenditure. Readings were performed over the day and night cycles using a Promethion cage system (n=3 per group).The bars and error graphs for body weight and cecum weight were represented by the mean and standard deviation, while metabolic output were represented by the median and interquartile ranges. Statistical comparisons for body weight, cecum weight and metabolic measures were performed using a linear mixed effects model, unpaired t-test and Mann-Whitney t-test, respectively, with significance determined at $p<0.05$.

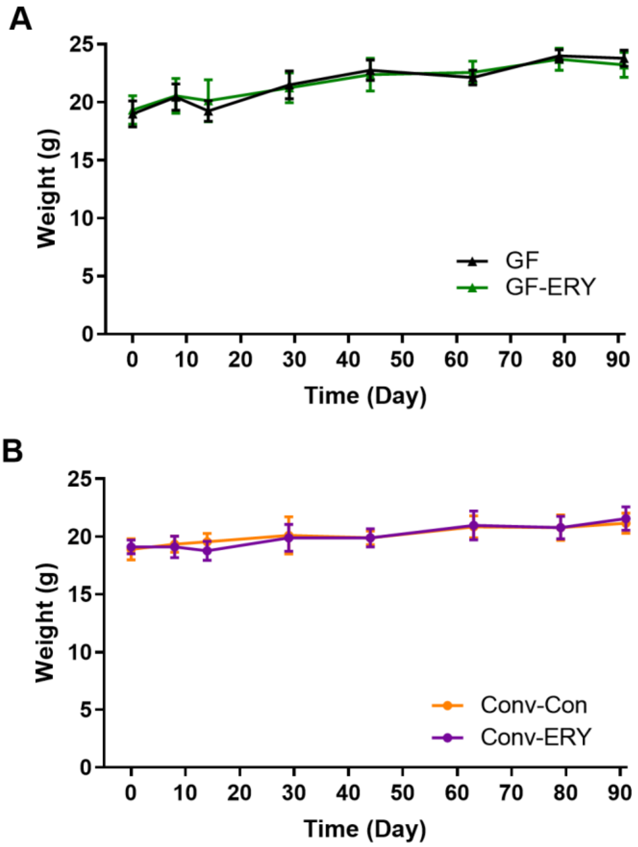

Figure S7. Physiological assessment of body weight of (A) germ-free mice that received erythromycin (20mg/kg) or water (n=7 per group), and (B) germ-free mice transplanted with erythromycin-associated microbiota or control microbiota (n=9 per group) over 90 days. Data are presented as the mean and the error bars represent the standard deviation. Statistical comparison between groups were performed using a linear mixed effects model, with significance determined at $p<0.05$.

REFERENCES

1. Burr, L. D., S. L. Taylor, A. Richard, V. Schreiber, S. Lingman, M. Martin, L. E. Papanicolas, J. M. Choo and G. B. Rogers (2022). Assessment of Long-Term Macrolide Exposure on the Oropharyngeal Microbiome and Macrolide Resistance in Healthy Adults and Consequences for Onward Transmission of Resistance. Antimicrob Agents Chemother 66, e0224621.

2. Wei, S., M. S. Mortensen, J. Stokholm, A. D. Brejnrod, J. Thorsen, M. A. Rasmussen, U. Trivedi, H. Bisgaard and S. J. Sorensen (2018). Short- and long-term impacts of azithromycin treatment on the gut microbiota in children: A double-blind, randomized, placebo-controlled trial. EBioMedicine 38, 265-272.

3. Doan, T., A. M. Arzika, K. J. Ray, S. Y. Cotter, J. Kim, R. Maliki, L. Zhong, Z. Zhou, T. C. Porco, B. Vanderschelden et al. (2017). Gut Microbial Diversity in Antibiotic-Naive Children After Systemic Antibiotic Exposure: A Randomized Controlled Trial. Clin Infect Dis 64, 1147-1153.

4. Nair, A. B. and S. Jacob (2016). A simple practice guide for dose conversion between animals and human. J Basic Clin Pharm 7, 27-31.

5. Truong, D. T., E. A. Franzosa, T. L. Tickle, M. Scholz, G. Weingart, E. Pasolli, A. Tett, C. Huttenhower and N. Segata (2015). MetaPhlAn2 for enhanced metagenomic taxonomic profiling. Nat Methods 12, 902-903.

6. Mobegi, F. M., L. E. Leong, F. Thompson, S. M. Taylor, L. R. Harriss, J. M. Choo, S. L. Taylor, S. L. Wesselingh, R. McDermott, K. L. Ivey et al. (2020). Intestinal microbiology shapes population health impacts of diet and lifestyle risk exposures in Torres Strait Islander communities. Elife 9.

7. Choo, J. M., C. D. Tran, N. D. Luscombe-Marsh, W. Stonehouse, J. Bowen, N. Johnson, C. H. Thompson, E. J. Watson, G. D. Brinkworth and G. B. Rogers (2021a). Almond consumption affects fecal microbiota composition, stool pH, and stool moisture in overweight and obese adults with elevated fasting blood glucose: A randomized controlled trial. Nutr Res 85, 47-59.

8. Choo, J. M. and G. B. Rogers (2021b). Establishment of murine gut microbiota in gnotobiotic mice. iScience 24, 102049.

9. Dey, N., V. E. Wagner, L. V. Blanton, J. Cheng, L. Fontana, R. Haque, T. Ahmed and J. I. Gordon (2015). Regulators of gut motility revealed by a gnotobiotic model of diet-microbiome interactions related to travel. Cell 163, 95-107.
